# Durable Disease Control in Primary Pulmonary Sarcomatoid Carcinoma Following Pneumonectomy

**DOI:** 10.3390/diagnostics15131718

**Published:** 2025-07-05

**Authors:** Cheng-Shiun Shiue, Chao-Chun Chang, Meng-Ta Tsai, Yu-Ning Hu

**Affiliations:** 1 Division of Cardiovascular Surgery, Department of Surgery, National Cheng Kung University Hospital, College of Medicine, National Cheng Kung University, Tainan 701, Taiwan; chensiun@gmail.com (C.-S.S.); dongsar@gmail.com (M.-T.T.); 2 Division of Thoracic Surgery, Department of Surgery, National Cheng Kung University Hospital, College of Medicine, National Cheng Kung University, Tainan 701, Taiwan; n048127@mail.hosp.ncku.edu.tw

**Keywords:** primary pulmonary sarcoma, pulmonary artery invasion, tumor embolus, PET/CT imaging, pneumonectomy, transcatheter arterial embolization, young adult lung cancer

## Abstract

We report a 26-year-old male presenting with a chronic cough and hemoptysis. Imaging revealed a large hypermetabolic mass in the left lower lung with the invasion of adjacent great vessels. A biopsy confirmed sarcomatoid carcinoma, a rare and aggressive form of primary pulmonary sarcoma. Due to vascular involvement, the patient underwent preoperative bronchial artery embolization followed by left pneumonectomy with pulmonary arterioplasty via median sternotomy. Postoperative recovery was uneventful. A two-year follow-up CT showed no recurrence. Primary pulmonary sarcomas are extremely rare, accounting for only 0.013–0.4% of lung malignancies, and are often diagnosed late due to nonspecific symptoms. This case highlights the importance of timely imaging, multidisciplinary planning, and aggressive surgical management in achieving long-term disease control, even in cases with extensive vascular invasion.

**Figure 1:**
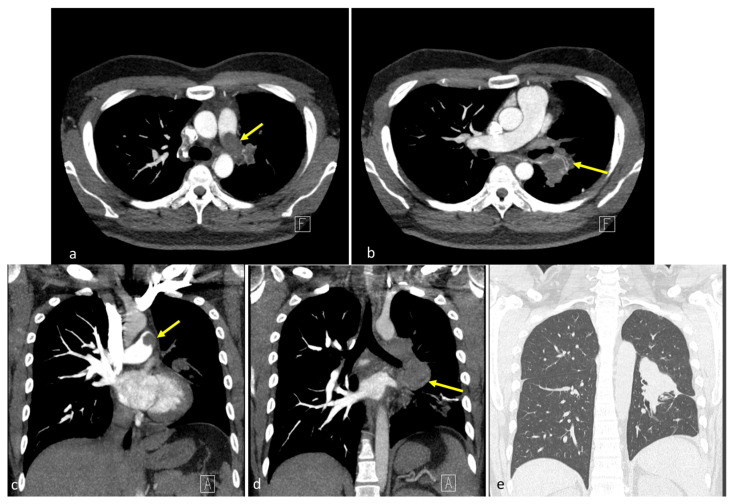
Preoperative computed tomography findings. (**a**,**b**) Coronal images revealed a 7.1 cm lobulated, heterogeneous mass spanning the upper left and lower lobes, extending medially into the pulmonary hilum (yellow arrows) [1,2,3]. The left pulmonary artery was encased with intraluminal lobulated filling defects, forming acute angles with the vessel wall, with suggested tumor thrombus. No pleural effusion or mediastinal shift was observed. (**c**,**d**) Axial sections showed a centrally located soft tissue mass with irregular margins involving the left hemithorax, with mild post-contrast enhancement and direct invasion into the left pulmonary artery. Intraluminal filling defects contiguous with the mass were identified. (**e**) Regarding lung window settings, the mass appeared as a dense, ill-defined opacity occupying the central left hemithorax, merging into the adjacent lung parenchyma. The letters “A” and “F” on the images represent standard orientation markers indicating anterior (A) and foot (F) directions, respectively.

**Figure 2:**
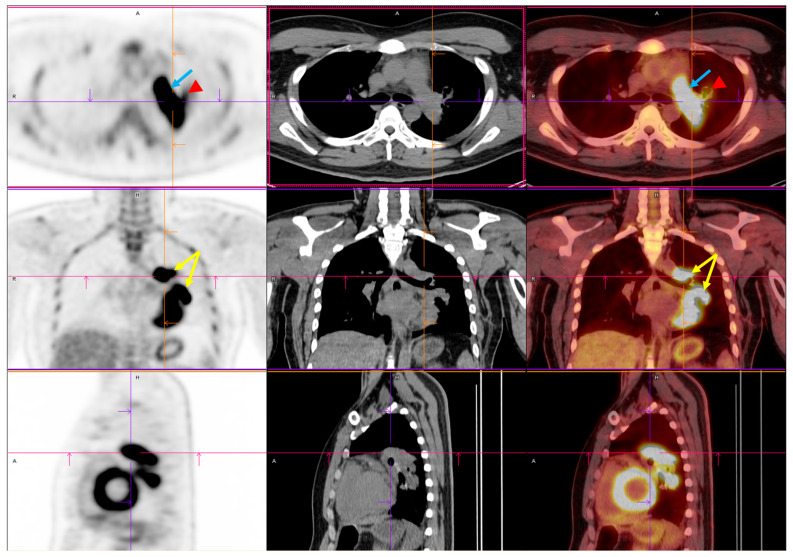
Preoperative PET/CT imaging. FDG PET/CT imaging demonstrated intense FDG uptake involving the left upper and lower lobes within the centrally located mass (yellow arrow) [4,5,6]. Upon initial imaging, the primary lesion exhibited a maximum standardized uptake value (SUVmax) of 14.52, with a delayed SUVmax of 21.08. This indicated significant metabolic activity and a high tumor glycolytic rate. The lesion’s metabolic borders corresponded closely to the soft tissue margins observed on CT, confirming its structural and functional malignancy. Crucially, increased FDG uptake was also identified within the left pulmonary artery (blue arrow) (SUVmax: 12.69), where a lobulated intraluminal filling defect had previously been observed on contrast-enhanced CT. The metabolic activity of the intravascular component mirrored that of the adjacent tumor, further supporting the diagnosis of a tumor thrombus rather than a bland thromboembolism [7]. This pattern of contiguous metabolic extension from the primary lung mass into the pulmonary vasculature strongly suggested direct vascular invasion. Mildly increased uptake was noted in the left hilar region (red arrowhead). This raised concerns about potential nodal involvement. Abbreviations: FDG, 18F-fluorodeoxyglucose; PET/CT, positron emission tomography/computed tomography; SUVmax, maximum standardized uptake value.

**Figure 3:**
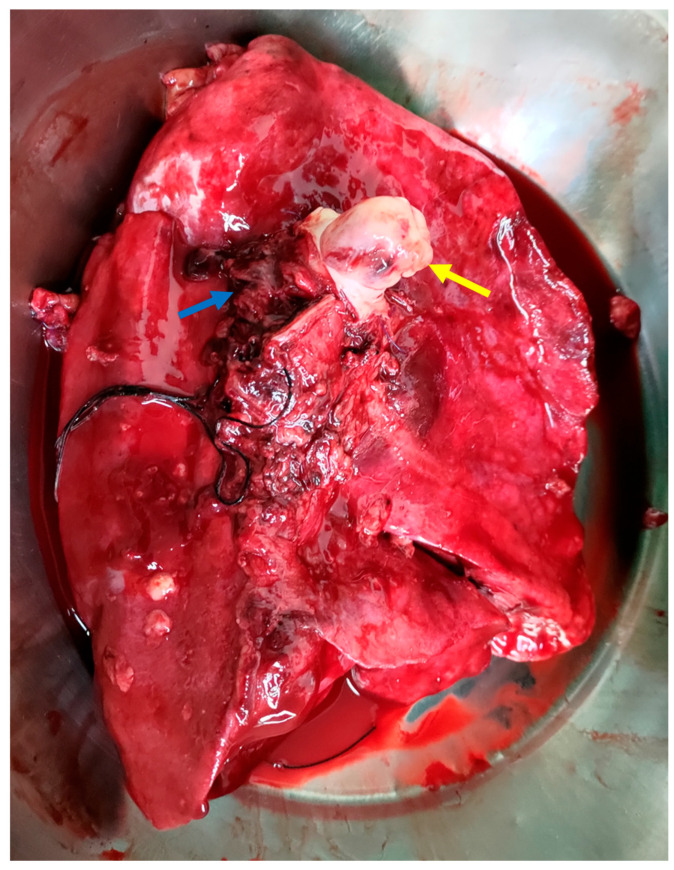
The intraoperative image shows tumor infiltration into the left pulmonary artery (yellow arrow), with clear involvement of the vessel wall, indicating vascular invasion. The left pulmonary veins (blue arrow) and the medially positioned trachea are also visible. This image highlights both the aggressiveness of the tumor and the complexity of the surgical resection required [7,8].

**Figure 4:**
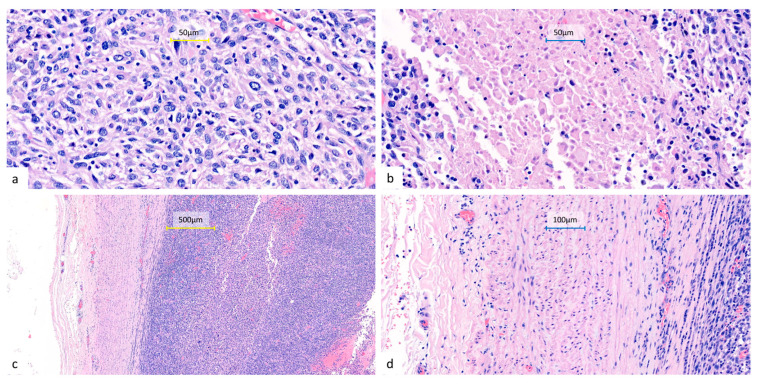
Representative histopathological features of the resected lung tumor. Hematoxylin and eosin-stained sections show sheets of undifferentiated epithelioid to spindle cells (**a**) (400×) with eosinophilic to amphophilic cytoplasm, marked nuclear pleomorphism, frequent mitoses, and focal necrosis (**b**) (400×). The tumor infiltrates the pulmonary artery with associated intraluminal tumor thrombi (**c**) (100×) (**d**) (200×). All surgical margins are free of tumor involvement, including a bronchial resection margin of 0.8 cm and a pulmonary artery resection margin of 1.0 cm from the nearest tumor invasion. No metastatic involvement can be seen in the sampled peribronchial lymph nodes (0/4) or pleural tissue.

**Figure 5:**
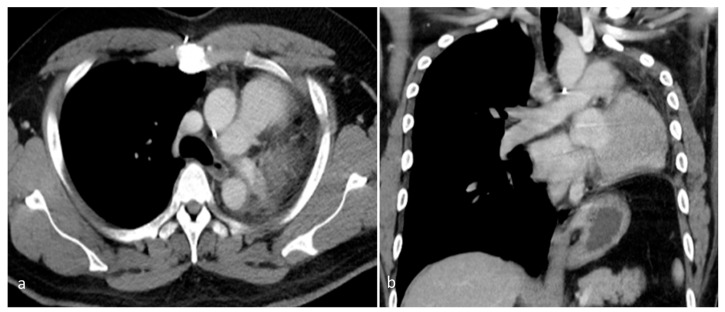
A contrast-enhanced chest CT, performed 2 years after surgery, revealed postoperative changes consistent with prior left pneumonectomy and the complete resection of the left pulmonary artery. (**a**) Axial view. (**b**) Coronal view. No residual or recurrent mass was apparent within the left hemithorax. The surgical bed appeared unremarkable, with no signs of local tumor recurrence or the abnormal enhancement of soft tissue. The right lung was well aerated, with no new parenchymal lesions or suspicious nodules. There was no evidence of contralateral lung involvement. No abnormally enlarged mediastinal or hilar lymph nodes were observed. These findings suggest a stable postoperative status with no radiological evidence of disease recurrence over 2 years after surgical resection.

## Data Availability

The data are available upon request from the authors. The data are not publicly available due to concerns regarding patient privacy and confidentiality.

## Abbreviations

The following abbreviations are used in this manuscript:

CT	Computed tomography
FDG	18F-fluorodeoxyglucose
PET	Positron emission tomography
SUVmax	Maximum standardized uptake value
